# Triglyceride-glucose index is associated with the occurrence and prognosis of cardiac arrest: a multicenter retrospective observational study

**DOI:** 10.1186/s12933-023-01918-0

**Published:** 2023-07-27

**Authors:** Yang Boshen, Zhu Yuankang, Zheng Xinjie, Li Taixi, Niu kaifan, Wang Zhixiang, Song Juan, Duan Junli, Li Suiji, Lu Xia, Shen Chengxing

**Affiliations:** 1 Department of Cardiology, Shanghai Sixth People’s Hospital, Shanghai Jiao Tong University School of Medicine, Shanghai, China; 2 Institute for Developmental and Regenerative Cardiovascular Medicine, Xinhua Hospital, School of Medicine, Shanghai Jiao Tong University, Shanghai, China; 3 Department of Gerontology, Xinhua Hospital, Shanghai Jiao Tong University School of Medicine, Shanghai, China; 4 Department of Respiratory Medicine, The Fourth Affiliated Hospital, College of Medicine, Zhejiang University, Yiwu, China; 5 Xiamen Cardiovascular Hospital, Xiamen University, Xiamen, China

**Keywords:** TyG index, Cardiac arrest, Intensive care unit, Prognosis, Hospital mortality

## Abstract

**Background:**

Triglyceride-glucose (TyG) index is an efficient indicator of insulin resistance and is proven to be a valuable marker in several cardiovascular diseases. However, the relationship between TyG index and cardiac arrest (CA) remains unclear. The present study aimed to investigate the association of the TyG index with the occurrence and clinical outcomes of CA.

**Methods:**

In this retrospective, multicenter, observational study, critically ill patients, including patients post-CA, were identified from the eICU Collaborative Research Database and evaluated. The TyG index for each patient was calculated using values of triglycerides and glucose recorded within 24 h of intensive care unit (ICU) admission. In-hospital mortality and ICU mortality were the primary clinical outcomes. Logistic regression, restricted cubic spline (RCS), and correlation analyses were performed to explore the relationship between the TyG index and clinical outcomes. Propensity score matching (PSM), overlap weighting (OW), and inverse probability of treatment weighting (IPTW) were adopted to balance the baseline characteristics of patients and minimize selection bias to confirm the robustness of the results. Subgroup analysis based on different modifiers was also performed.

**Results:**

Overall, 24,689 critically ill patients, including 1021 patients post-CA, were enrolled. The TyG index was significantly higher in patients post-CA than in those without CA (9.20 (8.72–9.69) vs. 8.89 (8.45–9.41)), and the TyG index had a moderate discrimination ability to identify patients with CA from the overall population (area under the curve = 0.625). Multivariate logistic regression indicated that the TyG index was an independent risk factor for in-hospital mortality (OR = 1.28, 95% CI: 1.03–1.58) and ICU mortality (OR = 1.27, 95% CI: 1.02–1.58) in patients post-CA. RCS curves revealed that an increased TyG index was linearly related to higher risks of in-hospital and ICU mortality (P for nonlinear: 0.225 and 0.271, respectively). Even after adjusting by PSM, IPTW, and OW, the TyG index remained a risk factor for in-hospital mortality and ICU mortality in patients experiencing CA, which was independent of age, BMI, sex, etc. Correlation analyses revealed that TyG index was negatively correlated with the neurological status of patients post-CA.

**Conclusion:**

Elevated TyG index is significantly associated with the occurrence of CA and higher mortality risk in patients post-CA. Our findings extend the landscape of TyG index in cardiovascular diseases, which requires further prospective cohort study.

**Supplementary Information:**

The online version contains supplementary material available at 10.1186/s12933-023-01918-0.

## Introduction

Triglyceride-glucose (TyG) index, a composite index derived from fasting blood glucose (FBG) and triglyceride (TG), was initially developed to be a good substitute for insulin resistance (IR) [1]. Interestingly, the value of TyG index in cardiovascular disorders has been gradually discovered in recent years. Researchers have demonstrated that TyG index was positively associated with the risk of heart failure (HF) [2], myocardial infarction (MI) [3], atrial fibrillation (AF) [4] and hypertension [5]. Elevated TyG levels were also reported to be related to the progression of coronary artery calcification [6] and a higher risk of arterial stiffness [7]. More importantly, extensive studies have discovered a strong association between TyG index and the prognosis of patients with cardiovascular diseases. For instance, Luo et al. found that MI patients after percutaneous coronary intervention with higher TyG levels showed a remarkably higher risk of mortality [8]. Similarly, Guo et al. showed that TyG index is positively correlated with the adverse outcomes of patients with HF and type 2 diabetes [9]. Overall, these findings indicated that TyG index was associated with many cardiovascular diseases and had a promising application prospect.

Cardiac arrest (CA), defined as sudden cessation of cardiac function, is a highly prevalent health condition with high morbidity and mortality rates worldwide [10–12]. Even worse, sudden cardiac death (SCD) caused by CA is the most frequent nontraumatic cause of death in the young population, and it is difficult to predict its occurrence [13, 14]. Although the prognosis of patients post-CA is gradually improving with advances in medical care, the mortality rate remains at a rather high level [11]. It was estimated that the current survival rate of patients post-CA was as low as about 10%, making it one of the primary killers in the United States [15]. Notably, CA is one of the most life-threatening complications of acute myocardial infarction [16], AF and severe HF [17, 18].

Many effective score systems have been developed to predict the clinical outcomes of patients post-CA, including CaRdiac Arrest Survival Score (CRASS), Cardiac Arrest Hospital Prognosis (CAHP) score [19, 20]. From an epidemiological perspective, coronary heart disease is a major risk factor for CA; therefore, they share some common clinical risk predictors such as diabetes mellitus, hypercholesterolemia, and obesity [21]. Among young individuals aged 5–34 years who experienced CA, 61% were reported to be obese or overweight [22]. Hyperglycemia is often observed in out-of-hospital patients after CA and is related to unfavorable clinical events [23]. Moreover, patients who undergo therapeutic hypothermia post-CA may have induction of insulin resistance (IR) and impairment of blood glucose homeostasis [24]. These findings indicated that glucose and lipid metabolism might be crucial to the pathogenesis and prognosis of patients post-CA, suggesting a potential unraveled relationship between TyG index and CA.

Up to now, the association between TyG index and CA has never been elucidated. The present study, with an aim to explore the association of TyG index with the occurrence and prognosis of CA, is the first study to report the unrecognized role of TyG index in the context of CA as an independent risk factor.

## Methods

### Data source

The study participants were identified from the eICU Collaborative Research Database [25], a multicenter database comprising the data of patients admitted to the intensive care unit (ICU) in the United States (US). This database contains high-quality data from over 200,000 admissions to the ICUs across 208 hospitals during 2014–2015 and is accessible to the public. This database includes diverse clinical data, such as information on demographic characteristics, vital signs, laboratory tests, disease diagnosis, and treatment approaches. The data were collected and normalized based on an effective electronic clinical management system. One of our authors was responsible for data extraction after gaining access to the database.

### Study design

This was a retrospective, observational, multicenter study. Among 200,859 ICU admission records in the eICU database, we excluded patients younger than 18 years old and those with missing values of TG and glucose within 24 h of admission, which resulted in 24,689 patients who had ICU admissions to the ICU. Among them, 1021 were diagnosed with CA at admission, identified based on ICD-9 codes recorded in the database. Moreover, patients who were in the post-CA period were divided into three groups based on tertiles of the TyG index calculated using TG and glucose within 24 h of admission or into two groups (lower TyG group and higher TyG group) for further analysis.

### Variable extraction

Baseline characteristics of patients, including demographic data, comorbidities, vital signs, clinical indicators, and treatment strategies, within 24 h of ICU admission were extracted to avoid potential confounders. Demographic parameters included age, sex, and body mass index (BMI). Vital signs such as heart rate (HR), systolic blood pressure (SBP), diastolic blood pressure (DBP), temperature, and saturation of peripheral oxygen (SpO_2_). MI, AF, chronic HF (CHF), chronic kidney disease (CKD), acute renal failure (ARF), diabetes, hypertension, and cardiogenic shock were included as comorbidities based on ICD-9 codes. Clinical indices included white blood cell (WBC) count, red blood cell (RBC) count, hemoglobin (HB), platelet count, creatinine, blood urea nitrogen (BUN), FBG, TG, TyG index, low-density lipoprotein (LDL), total cholesterol (TC), high-density lipoprotein (HDL), Acute Physiology Age Chronic Health Evaluation (APACHE) IV score and Acute Physiology Score (APS). Regarding treatment measures, the usage of enteral nutrition, dobutamine, dopamine, epinephrine, amiodarone, and statin were included.

Based on previous studies and the Utstein criteria [20, 26, 27], pre-hospital data such as bystander cardiopulmonary resuscitation (CPR), witnessed status, duration of CPR, initial rhythm were included to avoid potential bias. Arterial pH and PaCO_2_ measured within 24 h at ICU admission were also considered [28]. Specifically, duration of CPR was divided by 15 min, and ventricular fibrillation or pulseless ventricular tachycardia were defined as non-shockable initial rhythm [19]. We used the average value of a variable when it was recorded more than once within the first 24 h. Missing values were supplemented with multiple imputations. TyG index was calculated using the following formula: TyG index = ln [fasting TG (mg/dL) × fasting glucose (mg/dL)]/2, which was consistent with previous studies [29, 30].

### Clinical outcomes

Primary outcomes were defined as all-cause mortality in the ICU and the hospital. Length of stay (LOS) in the hospital and that in the ICU were the secondary outcomes. Glasgow Coma Scale (GCS) of patients on the first day of ICU admission was also included to reflect the patient’s neurological status.

### Statistical analysis

According to types of variables and distributions, the data assessment method and data presentation varied. Fisher’s exact or chi-square test was used to evaluate categorical variables, which were presented as numbers (percentages). Wilcoxon rank-sum test, Student’s t-test, or one-way analysis of variance was used for assessing continuous variables, which were presented as median (25th-75th percentiles) or as mean ± standard deviation.

Using logistic regression models, multivariate modeling of the associations between the TyG index and in-hospital mortality or ICU mortality was investigated. The multivariate regression models evaluated each potential confounder. Data were presented as odds ratio (OR) and 95% confidence interval (CI) to represent the effect of the TyG index on in-hospital or ICU mortality. Additionally, we used restricted cubic spline (RCS), with three knots being placed at the 25th, 50th, and 75th percentiles of the distribution to investigate the potential nonlinear relationships between the TyG index and in-hospital or ICU mortality.

After the patients post-CA were divided into the lower and higher TyG groups based on TyG index, propensity score matching (PSM) was performed. PSM is a common technique wherein the potential confounders are balanced in a study population and the reliability of the findings is assessed. Based on nearest neighbor matching, a 1:1 matching PSM analysis without replacement was conducted. In the current investigation, 0.02 was selected as the caliper width for PSM. In the PSM cohort, every possible confounder was considered. The results were obtained following PSM, with 199 patients being divided into two groups.

Overlap weighting (OW) and inverse probability of treatment weighting (IPTW) were also used to balance baseline attributes and to reduce selection bias. To weigh each patient, the propensity score (PS) was determined in IPTW, with the higher TyG group weight being 1/PS and the lower TyG group weight being 1/(1 − PS). Eventually, a standard population was obtained by applying standardized PS weighting to each patient. The bias between the higher TyG group and the lower TyG group tended to be consistent in the general population, which indicated that the difference in clinical outcomes between the two groups can be related to the varied TyG indices in each group. The advantage of the IPTW approach is that it can prevent sample size loss when compared to the straightforward 1:1 PSM. OW, a PS technique, was performed to simulate key characteristics of randomized controlled trials. In a nutshell, patients who were in post-CA and had higher TyG indices were weighed by the probability of having lower TyG indices (1 − PS), whereas patients who were in post-CA and had lower TyG indices were weighed by the probability of having higher TyG indices (PS).

Spearman and Pearson correlation analyses were used to explore the association between TyG index and continuous variables such as LOS, GCS, APS and APACHE IV.

Subgroup analysis was further conducted according to age, BMI, sex, CHF, cardiogenic shock, MI, AF, and diabetes to verify the prognostic value of the TyG index for in-hospital mortality and ICU mortality in each subgroup.

In this research, statistical analyses were conducted based on SPSS (version 23.0), Stata (version 14.0) and R software (version 4.1.2). A threshold of 0.05 was regarded as statistically significant for P-value.

## Results

### Elevated TyG index was observed in patients diagnosed with CA

**Fig. 1:**
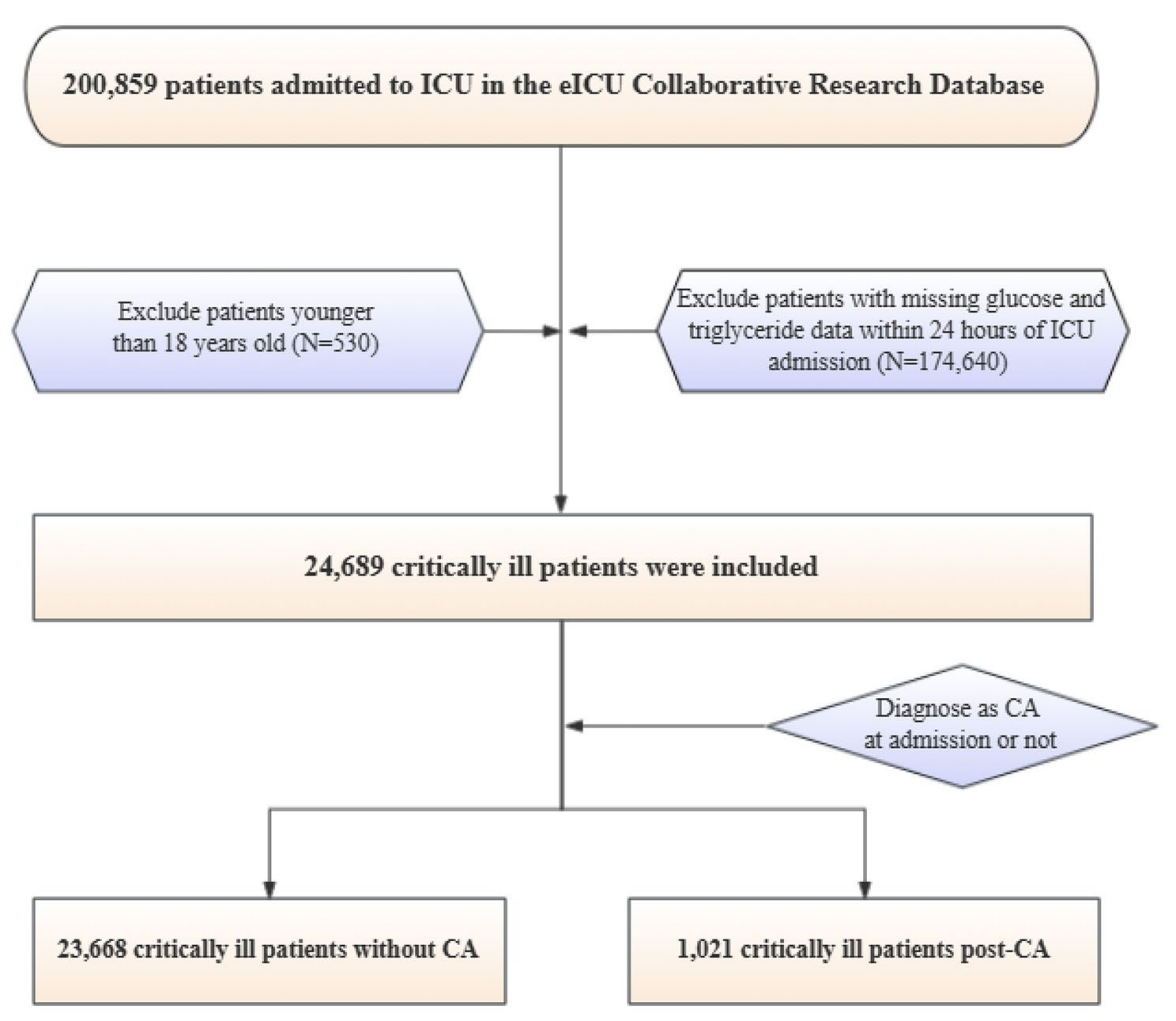
Study flowchart. ICU, intensive care unit; CA, cardiac arrest

The process of the participant recruitment is displayed as a flowchart in Fig. 1. Overall, 24,689 critically ill individuals admitted to ICUs were enrolled. Among them, 1021 were diagnosed with CA at admission. Data on baseline characteristics between patients post-CA and those who did not have CA are presented in Table 1.

**Table 1:** Baseline characteristics between critically ill patients without CA and with CA

	All critically ill patients (n = 24,689)	Patients without CA (n = 23,668)	Patients with CA (n = 1021)	P-value
**Demographic data**				
Age (years)	65(54–76)	65(54–76)	64(55–73)	0.078
Male (n (%))	14,313(58.0)	13,665(57.7)	648(63.5)	0.004
BMI	28.2(24.2–33.5)	28.2(24.1–33.5)	29.0(25.0–34.0)	<0.001
**Vital signs**				
HR (/min)	79(67.2–92.0)	79(68–92)	79(65–93)	0.739
SBP (mmHg)	124(108–141)	124(108–141)	115(100–135)	<0.001
DBP (mmHg)	68(58–78)	68(58–78)	64(55–75)	0.006
Temperature (℃)	36.8(36.5–37.1)	36.8(36.5–37.1)	36.6(34.6–37.1)	<0.001
SpO2 (%)	97(95–99)	97(95–99)	99(95–100)	0.002
**Comorbidities**				
MI (n (%))	4515(18.3)	4266(18.0)	249(24.4)	<0.001
AF (n (%))	2165(8.8)	2063(8.7)	102(10.0)	0.088
CHF (n (%))	2078(8.4)	1970(8.3)	108(10.6)	0.007
CKD (n (%))	1009(4.1)	951(4.0)	58(5.7)	0.005
ARF (n (%))	2173(8.8)	1940(8.2)	233(22.8)	<0.001
Diabetes (n (%))	2777(11.2)	2651(11.2)	126(12.3)	0.265
Hypertension (n (%))	4747(19.2)	4614(19.5)	133(13.0)	<0.001
Cardiogenic shock (n (%))	413(1.7)	291(1.2)	122(11.9)	<0.001
**Clinical indices**				
RBC (M/mcl)	4.1(3.5–4.5)	4.1(3.5–4.5)	4.1(3.5–4.6)	0.404
WBC (K/mcl)	10.0(7.1–14.0)	9.8(7.1–13.8)	13.2(9.9–19.0)	0.796
Platelet (K/mcl)	200(157–250)	201(157–250)	197.0(148.0-248.5)	0.110
HB (g/dL)	12.2(10.4–13.6)	12.2(10.5–13.6)	12.1(10.2–13.8)	0.592
Creatinine (mg/dL)	0.97(0.74–1.40)	0.96(0.74–1.38)	1.26(0.88–2.10)	<0.001
BUN (mg/dL)	17.0(12.0–27.0)	17.0(12.0–27.0)	22.0(15.0–34.0)	<0.001
FBG (mmol/L)	129.5(108.0-168.0)	128.5(107.5–166.0)	165.3(133.5–215.0)	<0.001
Triglyceride (mg/dL)	110.0(76.5–165.0)	110.0(76.0-164.0)	114.0(79.2–171.0)	<0.001
Tyg-index	8.90(8.46–9.42)	8.89(8.45–9.41)	9.20(8.72–9.69)	<0.001
TC (mg/dL)	150(120–184)	151(120–185)	136.0(107.4-166.3)	<0.001
HDL (mg/dL)	40.0(31.0-50.5)	40.0(31.0–51.0)	37.0(28.8–47.0)	<0.001
LDL (mg/dL)	82(58–112)	83(58–112)	71.0(47.6–96.9)	<0.001
GCS Verbal score Motor score Eyes score	15(12–15) 5(4–5) 6(6–6) 4(3–4)	15(13–15) 5(4–5) 6(6–6) 4(3–4)	7(3–14) 1(1–4) 4(1–6) 1(1–4)	<0.001 <0.001 <0.001 <0.001
APS	35(24–51)	35(24–50)	79(47–108)	<0.001
APACHE IV	48(34–65)	47(34–64)	91(60–122)	<0.001
**Treatment measures**				
Dobutamine (n (%))	146(0.6)	127(0.5)	19(1.9)	<0.001
Dopamine (n (%))	439(1.8)	329(1.4)	110(10.8)	<0.001
Epinephrine (n (%))	151(0.6)	95(0.4)	56(5.5)	<0.001
Norepinephrine (n (%))	1524(6.2)	1211(5.1)	313(30.7)	<0.001
Amiodarone (n (%))	379(1.5)	352(1.5)	27(2.6)	0.003
Statin (n (%))	475(1.9)	460(1.9)	15(1.5)	0.280

CA, cardiac arrest; BMI, body mass index; HR, heart rate; SBP, systolic blood pressure; DBP, diastolic blood pressure; SpO2, saturation of peripheral oxygen; MI, myocardial infarction; AF, atrial fibrillation; CHF, chronic heart failure; CKD, chronic kidney disease; ARF, acute renal failure; RBC, red blood cell; WBC, white blood cell; HB, hemoglobin; BUN, blood urea nitrogen ; FBG, fast blood glucose; TyG index, triglyceride-glucose index; TC, total cholesterol; HDL, high density lipoprotein; LDL, low density lipoprotein; GCS, Glasgow Coma Scale; APS, Acute Physiology Score; APACHE IV, Acute Physiology Age Chronic Health Evaluation IV.

Interestingly, patients post-CA had a significantly higher TyG index than those who did not have CA (9.20 (8.72–9.69) vs. 8.89 (8.45–9.41)). Then, we investigated the association between the TyG index and the risk of being diagnosed with CA using logistic regression analysis. As displayed in Table 2, unadjusted model 1 revealed that the TyG index was a risk factor for patients who were diagnosed with CA (OR = 1.37, 95% CI: 1.29–1.47). After adjusting for age, sex, and BMI, the association remained stable (OR = 1.37, 95% CI: 1.28–1.46). As shown in model 3, the TyG index was a significant independent risk factor for a CA diagnosis (OR = 1.57, 95% CI: 1.43–1.72) after adjusting for all potential confounders including age, sex, BMI, CHF, cardiogenic shock, ARF, AF, diabetes, hypertension, CKD, MI, platelet, SBP, DBP, HR, WBC, HDL, LDL, TC, RBC, BUN, creatinine, HB, temperature, SpO_2_, norepinephrine, epinephrine, dobutamine, dopamine, statin, and amiodarone.

**Table 2:** Logistic regression analysis to explore odds ratios of TyG index for patients being diagnosed with CA

TyG index	ORs for patients diagnosed as CR	P value
Model 1	1.37(1.29–1.47)	<0.001
Model 2	1.37(1.28–1.46)	<0.001
Model 3	1.57(1.43–1.72)	<0.001

Model 1 was unadjusted

Model 2 was adjusted by age, gender, and BMI.

Model 3 was adjusted by age, gender, BMI, CHF, Cardiogenic shock, ARF, AF, diabetes, hypertension, CKD, MI, Platelet, SBP, DBP, HR, WBC, HDL, LDL, TC, RBC, BUN, Creatinine, HB, temperature, SpO2, Norepinephrine, Epinephrine, Dobutamine, Dopamine, Statin, Amiodarone

TyG index, triglyceride glucose index; CA, cardiac arrest; OR, odds ratio

**Fig. 2:**
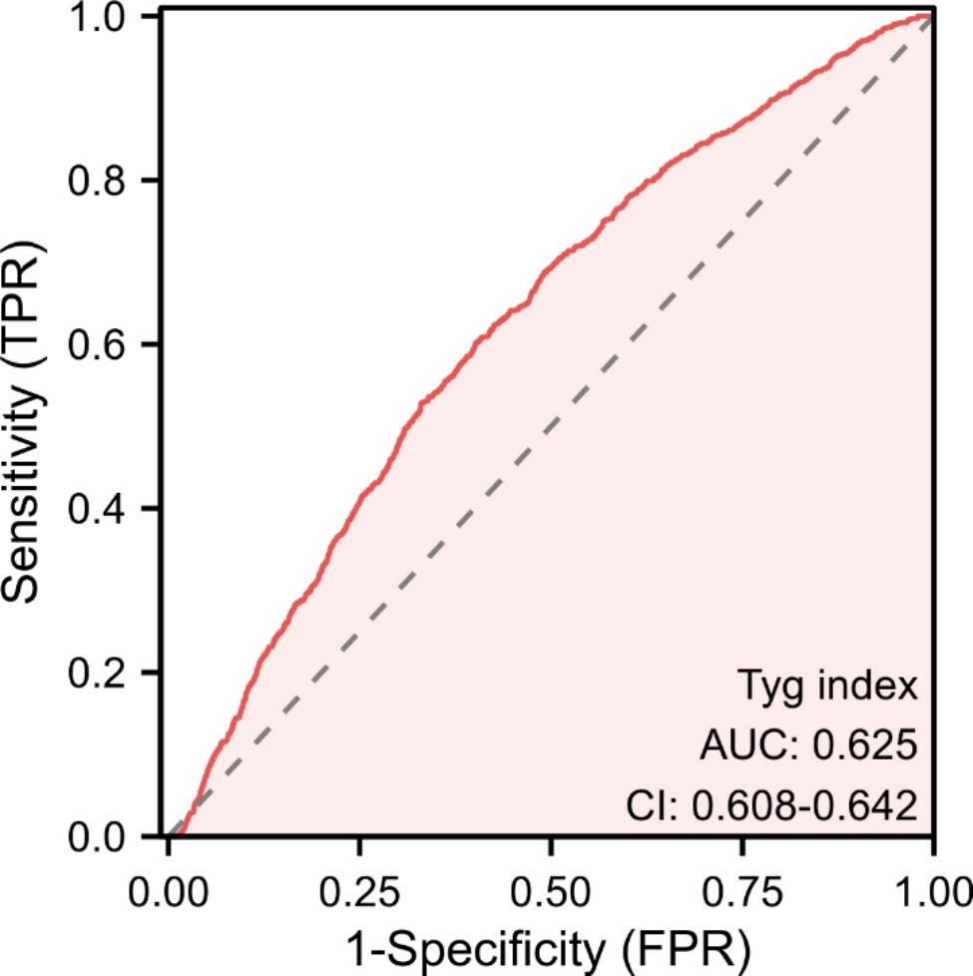
ROC curve of TyG index for identifying patients diagnosed with CA from overall critically ill patients. TyG index, triglyceride glucose index; CA, cardiac arrest; ROC, Receiver operating characteristic; TPR, true positive rate; FPR, false positive rate

Additionally, as shown in Fig. 2, the receiver operating characteristic (ROC) curve revealed that the TyG index had better performance in identifying patients post-CA from the overall critically ill patients (area under the curve: 0.625 (95% CI: 0.608–0.642)) than age, sex, BMI, LDL, HDL, and TC (Table S1).

Taken together, these results proved the association between elevated TyG levels and CA diagnosis, indicating that TyG has the ability to be a useful indicator in predicting CA occurrence.

### Baseline characteristics and clinical outcomes of patients in different TyG tertiles post-CA

We further explored the relationship of the TyG index with clinical outcomes in patients post-CA. As shown in Table S2, among 1021 patients post-CA, 642 survived at hospital discharge, whereas 379 patients died during the hospital stay. Notably, non-survivors had a significantly higher TyG index than survivors (9.28 (8.81–9.86) vs. 9.13 (8.68–9.57)). We further divided patients post-CA into three groups according to tertiles of the TyG index (Table 3). Compared with patients post-CA in the lowest group, those in the highest tertile group were generally younger, predominantly male, and had higher BMI. No significant differences were found in vital signs except for higher HR in the highest tertile group. As for comorbidities, patients post-CA with the highest TyG index had a higher incidence of ARF, while no significant differences were observed in MI, AF, CHF, CKD, diabetes, hypertension, cardiogenic shock, and non-shockable rhythm. Patients in the highest group had higher RBC, WBC, PaCO_2_, and platelet counts; HB; FBG; TG; TC; LDL; APS and APACHE IV scores but lower levels of HDL and arterial pH. Notably, no significant differences were found regarding treatment measures, including the usage of amiodarone, dobutamine, dopamine, epinephrine, statin, and enteral nutrition. Moreover, similar pre-hospital data such as bystander CPR and unwitnessed status were observed, except for the duration of CPR lower than 15 min.

**Table 3:** Baseline characteristics of patients post-CA grouped according to TyG index tertile

TyG index		Tertile 1 (n = 340)	Tertile 2 (n = 341)	Tertile 3 (n = 340)	P-value
**Demographic data**					
Age (years)		65(56–76)	65(56–74)	62(53–69)	<0.001
Male (n (%))		232(68.2)	205(60.1)	211(62.1)	0.072
BMI		27.3(23.7–31.9)	28.6(24.9–32.9)	31.0(26.6–36.3)	<0.001
**Vital signs**					
HR (/min)		78(63–91)	78(64–93)	81(68–96)	0.012
SBP (mmHg)		116(101–135)	114(99–134)	114(100–137)	0.928
DBP (mmHg)		65(56–75)	63(54–73)	65(55–77)	0.219
Temperature (℃)		36.6(35.7–37.1)	36.5(34.5–37.0)	36.5(34.3–37.3)	0.226
SpO2 (%)		99(95–100)	99(96–100)	99(95–100)	0.079
**Comorbidities**					
MI (n (%))		90(26.5)	88(25.8)	71(20.9)	0.179
AF (n (%))		34(10.0)	35(10.3)	33(9.7)	0.971
CHF (n (%))		42(12.4)	30(8.8)	36(10.6)	0.321
CKD (n (%))		18(5.3)	21(6.2)	19(5.6)	0.885
ARF (n (%))		61(17.9)	70(20.5)	102(30.0)	<0.001
Diabetes (n (%))		33(9.7)	45(13.2)	48(14.1)	0.182
Hypertension (n (%))		43(12.6)	49(14.4)	41(12.1)	0.648
Cardiogenic shock (n (%))		35(10.3)	35(10.3)	52(15.3)	0.066
Non-shockable rhythm (n (%))		98(28.8)	79(23.2)	78(22.9)	0.133
**Clinical indices**					
RBC (M/mcl)		4.0(3.5–4.5)	4.0(3.4–4.5)	4.2(3.6–4.7)	0.002
WBC (K/mcl)		11.7(9.0-16.3)	13.0(10.0-18.5)	15.6(10.8–21.9)	<0.001
Platelet (K/mcl)	186(140–242)		203(155–254)	203(151–250)	0.020
HB (g/dL)		11.9(10.0-13.4)	11.8(10.0-13.5)	12.6(10.3–14.2)	0.002
Creatinine (mg/dL)		1.10(0.84–1.80)	1.20(0.86–2.05)	1.49(0.99–2.25)	0.066
BUN (mg/dL)		20.0(14.0–32.0)	21.0(15.0–32.0)	24.0(17.0–37.0)	0.203
FBG (mg/dL)		135.0(116.2-163.8)	162.3(136.6-200.8)	220.7(171.4-278.9)	<0.001
Triglyceride (mg/dL)		70.0(56.0–87.0)	118.0(95.5-139.3)	197.0(152.0-261.3)	<0.001
Tyg-index		8.51(8.24–8.72)	9.20(9.04–9.32)	9.91(9.70–10.30)	<0.001
TC (mg/dL)		126.3(98.4-158.9)	136.0(110.1-164.3)	144.0(113.0-176.2)	<0.001
HDL (mg/dL)		41.5(32.0–52.0)	38.0(30.0-46.8)	32.0(25.3–43.0)	<0.001
LDL (mg/dL)		66.0(44.1–93.8)	71.4(49.8–96.0)	78.0(49.3–99.0)	0.027
Arterial pH		7.35(7.28–7.41)	7.36(7.29–7.43)	7.31(7.23–7.40)	0.002
PaCO2 (mmHg)		41(33–47)	38(33–46)	40(33–46)	0.026
APS		71(38–101)	76(47–103)	94(62–120)	<0.001
APACHE IV		85(51–114)	89(60–117)	104(72–129)	<0.001
**Treatment measures**					
Bystander CPR (n (%))		134(39.4)	117(34.3)	113(33.2)	0.199
CPR>15 min (n (%)) CPR<15 min (n (%))		26(7.6) 108(31.8)	26(7.6) 92(27.0)	37(10.9) 78(22.9)	0.223 0.035
Unwitnessed status (n (%))		13(38.2)	8(2.3)	13(3.8)	0.463
Enteral nutrition (n (%))		2(0.6)	3(0.9)	0(0)	0.246
Amiodarone (n (%))		10(2.9)	5(14.7)	12(3.5)	0.224
Dobutamine (n (%))		7(2.1)	7(2.1)	5(1.5)	0.808
Dopamine (n (%))		39(11.5)	39(11.4)	32(9.4)	0.611
Epinephrine (n (%))		18(5.3)	18(5.3)	20(5.9)	0.925
Statin (n (%))		3(0.9)	8(2.3)	4(1.2)	0.244

CA, cardiac arrest; BMI, body mass index; HR, heart rate; SBP, systolic blood pressure; DBP, diastolic blood pressure; SpO2, saturation of peripheral oxygen; MI, myocardial infarction; AF, atrial fibrillation; CHF, chronic heart failure; CKD, chronic kidney disease; ARF, acute renal failure; RBC, red blood cell; WBC, white blood cell; HB, hemoglobin; BUN, blood urea nitrogen ; FBG, fast blood glucose; TyG-index, triglyceride-glucose index; TC, total cholesterol; HDL, high density lipoprotein; LDL, low density lipoprotein; APS, Acute Physiology Score; APACHE IV, Acute Physiology Age Chronic Health Evaluation IV. TPN, total parenteral nutrition; CPR, cardiopulmonary resuscitation

The clinical outcomes of patients post-CA in the three groups are presented in Table 4. Patients with a higher TyG index had significantly higher in-hospital and ICU mortality rates. However, no significant differences were observed in hospital and ICU LOS among three groups.

**Table 4:** Clinical outcomes of patients post-CA grouped according to TyG index tertile

Clinical outcomes	Tertile 1 (n = 340)	Tertile 2 (n = 341)	Tertile 3 (n = 340)	P-value
**Primary outcomes**				
In-hospital mortality (n (%))	104(30.6)	119(34.9)	156(45.9)	<0.001
ICU mortality (n (%))	86(25.3)	100(29.3)	128(37.6)	0.002
**Secondary outcomes**				
Hospital LOS (days)	6.0(2.9–12.1)	6.0(3.2–11.7)	5.6(2.9–12.7)	0.398
ICU LOS (days)	2.9(1.4–6.5)	3.3(1.6-6.0)	3.5(1.7–7.1)	0.053

CA, cardiac arrest; TyG index, triglyceride glucose index; ICU, intensive care unit; LOS, length of stay

Tertile1: TyG index ≤ 8.89; Tertile2: 8.89 < TyG index ≤ 9.49; Tertile3: TyG index > 9.49

**Fig. 3:**
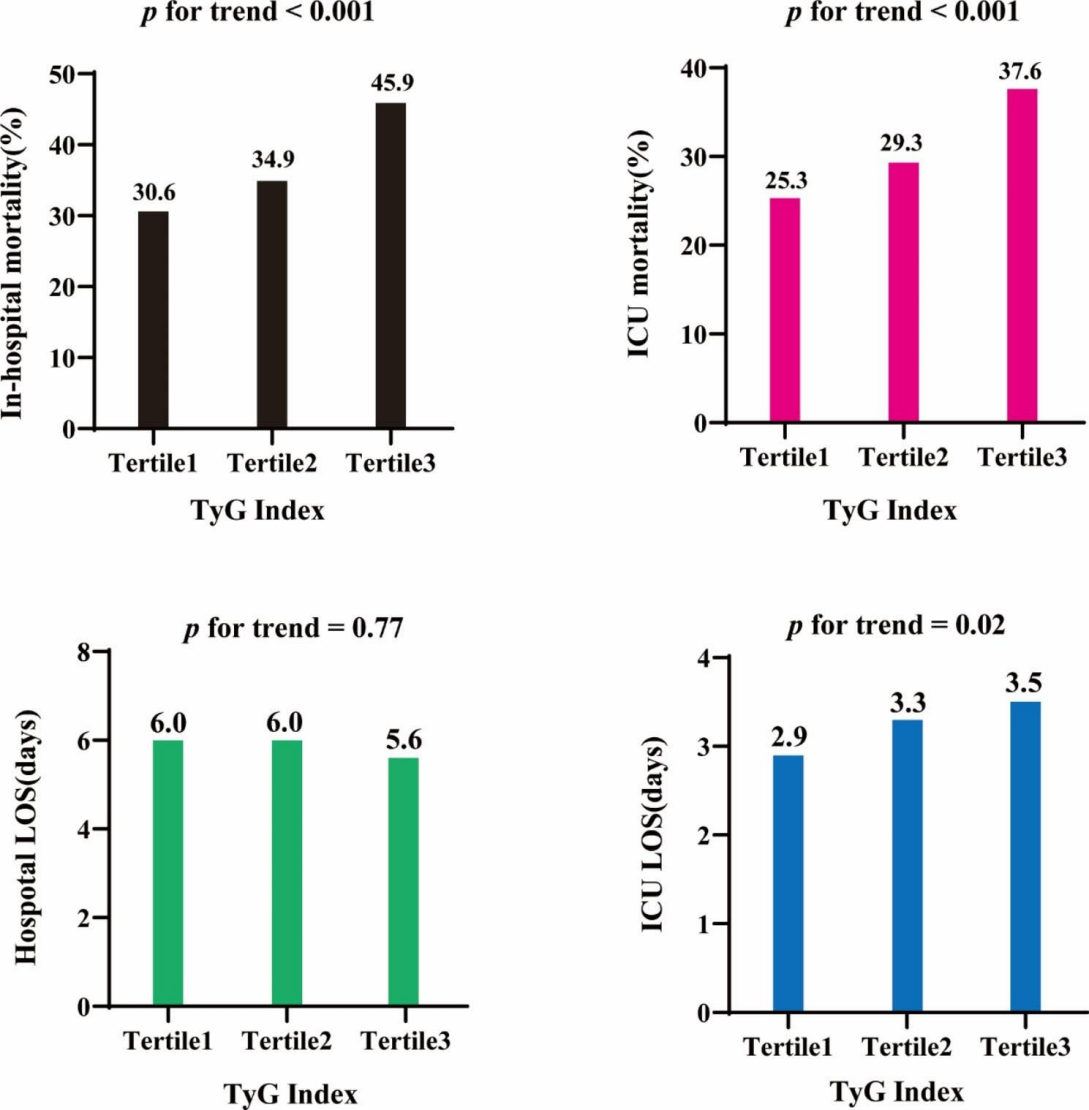
Primary and secondary clinical outcomes based on tertiles of TyG index in patients post-CA. (A) In-hospital mortality. (B) ICU mortality. (C) Hospital LOS. (D) ICU LOS. TyG index, triglyceride-glucose index; LOS, length of stay; ICU, intensive care unit; CA, cardiac arrest. Tertile1: TyG index ≤ 8.89; Tertile2: 8.89 < TyG index ≤ 9.49; Tertile3: TyG index > 9.49

Then, we performed a trend analysis for clinical outcomes (Fig. 3). Interestingly, we discovered a significantly increasing trend for in-hospital mortality and ICU mortality, with the P for trend being <0.001 with an increased tertile of the TyG index. An increasing trend of LOS in the ICU was observed among the three groups, with the P for trend being 0.02, while no trend was seen for LOS in the hospital (P for trend = 0.77). Taken together, these results indicate that the TyG index has the potential to be related to the clinical outcomes of patients experiencing CA.

### Associations between TyG index and clinical outcomes in patients post-CA

To further evaluate whether the TyG index is a risk factor for in-hospital mortality and ICU mortality, multivariate logistic regression analysis was used. As shown in Table 5, in-hospital mortality risk (OR = 1.36, 95% CI:1.14–1.62) and ICU mortality risk (OR = 1.38, 95% CI:1.15–1.65) were significantly higher in patients post-CA in the highest tertile than in those in the lowest tertile in unadjusted model 1. After adjusting for age, sex, and BMI, the association remained stable, with an OR of 1.46 (95% CI:1.22–1.75) for in-hospital mortality and an OR of 1.46 (95% CI:1.21–1.76) for ICU mortality. The results were robust in the fully adjusted model 3, including age, gender, BMI, CHF, Cardiogenic shock, ARF, AF, diabetes, hypertension, CKD, MI, Platelet, SBP, DBP, HR, WBC, HDL, LDL, TC, RBC, BUN, Creatinine, HB, temperature, SpO2, epinephrine, dobutamine, dopamine, amiodarone, statin, non-shockable rhythm, arterial pH, bystander CPR, CPR>15 min, CPR<15 min, unwitnessed status, enteral nutrition, and the OR of the highest tertile for in-hospital mortality was 1.32 95% CI:1.08–1.62) while the OR of the highest tertile for ICU mortality was 1.27 (95% CI = 1.05–1.53) compared with the lowest tertile. Moreover, the risk of in-hospital mortality and ICU mortality showed a tendency to increase with the increased tertile of the TyG index, with all P-values for the trend being lower than 0.05.

**Table 5:** Odd ratios of TyG index for in-hospital and ICU mortality in patients post-CA

TyG index	OR (95%CI)		
**In-hospital mortality**	Model 1	Model 2	Model 3
Per 1 Unit increase	1.35(1.13–1.60)	1.40(1.17–1.67)	1.28(1.03–1.58)
Tertile 1	Reference	Reference	Reference
Tertile 2	1.22(0.88–1.68)	1.20(0.87–1.66)	1.23(0.83–1.82)
Tertile 3 P for trend	1.36(1.14–1.62) <0.001	1.46(1.22–1.75) <0.001	1.32(1.08–1.62) 0.001
**ICU mortality**			
Per 1 Unit increase	1.36(1.14–1.62)	1.44(1.19–1.73)	1.27(1.02–1.58)
Tertile 1	Reference	Reference	Reference
Tertile 2	1.23(0.87–1.72)	1.23(0.88–1.74)	1.29(0.88–1.93)
Tertile 3 P for trend	1.38(1.15–1.65) 0.001	1.46(1.21–1.76) <0.001	1.27(1.05–1.53) 0.005

Model 1 was unadjusted

Model 2 was adjusted by age, gender, BMI.

Model 3 was adjusted by age, gender, BMI, CHF, Cardiogenic shock, ARF, AF, diabetes, hypertension, CKD, MI, Platelet, SBP, DBP, HR, WBC, HDL, LDL, TC, RBC, BUN, Creatinine, HB, temperature, SpO2, epinephrine, dobutamine, dopamine, amiodarone, statin, non-shockable rhythm, arterial pH, bystander CPR, CPR>15 min, CPR<15 min, unwitnessed status, enteral nutrition

Tertile1: TyG index ≤ 8.89; Tertile2: 8.89 < TyG index ≤ 9.49; Tertile3: TyG index > 9.49

TyG index, triglyceride glucose index; ICU, intensive care unit; CA, cardiac arrest; OR, odds ratio

Additionally, Pearson’s and Spearman’s analyses were used for evaluating the correlation between TyG index and LOS in the hospital and ICU, HDL, LDL, TC, and APS and APACHE IV scores, and a significant correlation was noted between TyG index and APS and TC (Table S3). Multiple linear regression analysis further confirmed that the TyG index was positively associated with APS and APACHE IV scores (β = 1.02; Table S4). These results confirmed that the TyG index was strongly associated with adverse clinical outcomes in patients post-CA as an independent risk factor, especially for in-hospital and ICU mortality.

### Patients post-CA with elevated TyG index had higher risk of mortality

**Fig. 4:**
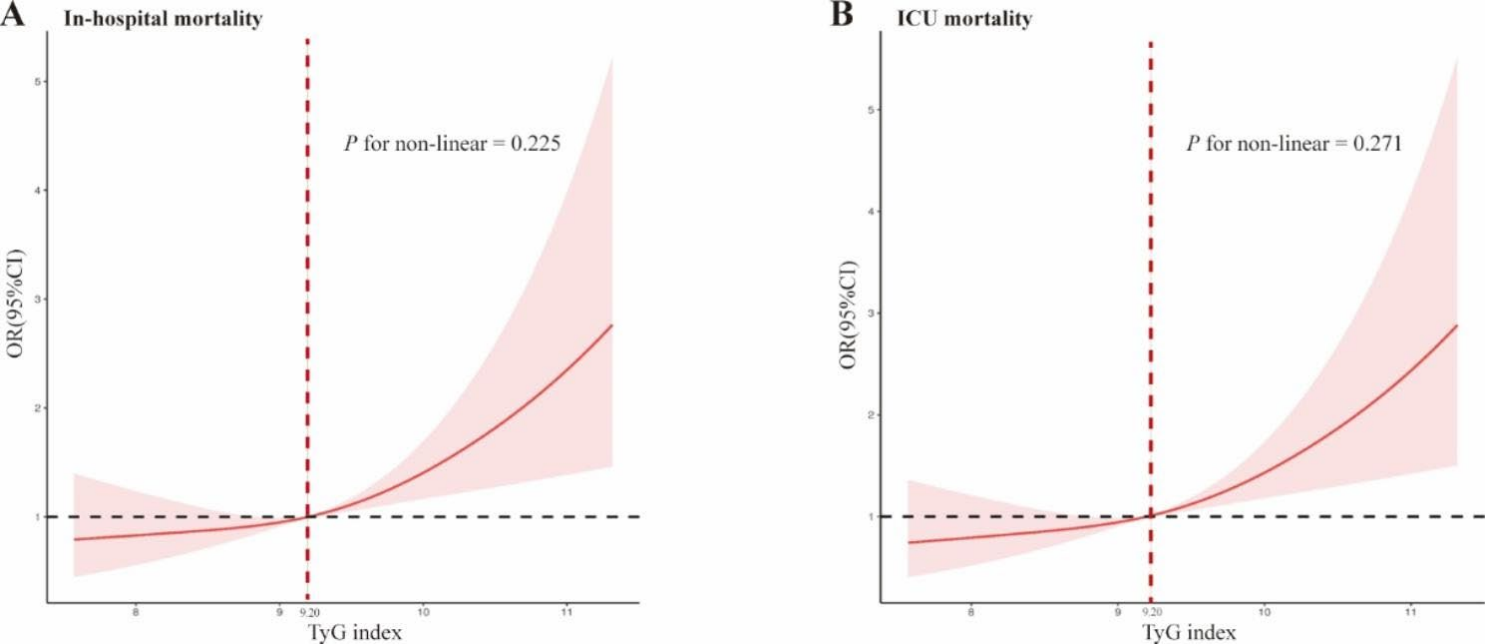
RCS curves of TyG index with in-hospital mortality and ICU mortality in patients post-CA. (A) RCS curves of TyG index with in-hospital mortality (B) RCS curves of TyG index with ICU mortality. TyG-index, triglyceride-glucose index; ICU, intensive care unit; RCS, restricted cubic spline

The RCS results were shown in Fig. 4 after adjusting for age, sex, and BMI. An increased TyG index was linearly associated with higher in-hospital and ICU mortality risk (P for nonlinear = 0.225 and 0.271), especially in patients with a TyG index > 9.20.

Based on the results of RCS, we further divided the patients post-CA into two groups according to the TyG index threshold of 9.20, and baseline characteristics of the two groups were shown in Table S5. To balance potential confounders and minimize selection bias, we used 1:1 PSM, IPTW, and OW. The baseline characteristics in the PSM-adjusted cohort were presented in Table 6, with 199 patients being included in each group, while the baseline characteristics in IPTW and OW-adjusted cohort were shown in Table S6. All the potential confounders were balanced after PSM, including APS, APACHE IV scores and GCS, and most confounders were balanced after IPTW and OW.

**Table 6:** Baseline characteristics in the matched cohort post PSM analysis

	Overall PSM-matched cohort(n = 398)	Lower TyG-index group (n = 199)	Higher TyG-index group (n = 199)	P-value
**Demographic data**				
Age (years)	65(55–74)	65(54–74)	65(56–73)	1.000
Male (n (%))	246(61.8)	120(60.3)	126(63.3)	0.606
BMI	28.2(24.4–32.5)	28.0(24.2–32.7)	28.4(24.5–32.5)	0.749
**Vital signs**				
HR (/min)	80(67–96)	82(67–97)	79(66–96)	0.820
SBP (mmHg)	113(98–135)	112(98–133)	114(97–138)	0.750
DBP (mmHg)	63(54–73)	65(56–76)	62(53–72)	0.011
Temperature (℃)	36.4(34.0-37.2)	36.4(33.8–37.2)	36.6(34.1–37.3)	0.488
SpO2	99(95–100)	99(95–100)	99(96–100)	0.893
**Comorbidities**				
MI (n (%))	83(20.9)	43(21.6)	40(20.1)	0.805
AF (n (%))	36(9.0)	18(9.0)	18(9.0)	1.000
CHF (n (%))	41(10.3)	18(9.0)	23(11.6)	0.510
CKD (n (%))	31(5.3)	11(5.5)	10(5.0)	1.000
ARF (n (%))	104(26.1)	50(25.1)	54(27.1)	0.732
Diabetes (n (%))	47(11.8)	24(12.1)	23(11.6)	1.000
Hypertension (n (%))	50(12.6)	25(12.6)	25(12.6)	1.000
Cardiogenic shock (n (%))	55(13.8)	28(14.1)	27(13.6)	1.000
Non-shockable rhythm (n (%))	74(18.6)	34(17.1)	40(20.1)	0.520
**Clinical indices**				
RBC (M/mcl)	4.0(3.3–4.5)	4.0(3.3–4.6)	3.9(3.3–4.4)	0.454
WBC (K/mcl)	13.9(10.0-19.3)	13(9.9–18.5)	14.2(10.1–20.0)	0.918
Platelet (K/mcl)	192(140–244)	190(141–256)	193(139–242)	0.272
HB (g/dL)	11.8(9.9–13.6)	11.9(10.0-13.9)	11.7(9.7–13.3)	0.414
Creatinine (mg/dL)	1.43(0.94–2.27)	1.34(0.92–2.28)	1.47(0.97–2.27)	0.725
BUN (mg/dL)	25.5(18.0–36.0)	25.0(17.0–36.0)	26.0(19.0–36.0)	0.735
FBG (mg/dL)	156.0(121.0-233.3)	140.0(115.0-179.0)	201.0(131.0-277.0)	< 0.001
Triglyceride (mg/dL)	109.0(81.0-151.0)	86.0(69.0-106.0)	146.0(112.0-208.0)	< 0.001
Tyg-index	9.20(8.80–9.61)	8.80(8.47–8.98)	9.60(9.35–10.08)	< 0.001
TC (mg/dL)	127.4(102.3-153.5)	133.8(108.0-161.0)	125.0(97.0-147.0)	0.093
HDL (mg/dL)	37.0(28.0–48.0)	37.0(29.0–47.0)	36.0(27.0–48.0)	0.319
LDL (mg/dL)	65.9(45.0-88.5)	68.0(47.0-92.8)	62.0(42.1–86.0)	0.140
Arterial pH	7.35(7.25–7.42)	7.35(7.26–7.41)	7.35(7.24–7.42)	0.868
PaCO_2_ (mmHg)	40(33–47)	40(33–47)	40(34–46)	0.618
APS	88(66–111)	86(66–109)	89(64–115)	0.608
APACHE IV	100(76–125)	99(76–122)	102(75–127)	0.552
GCS Verbal score Motor score Eyes score	3(3–10) 1(1–1) 1(1–5) 1(1–3)	4(3–10) 1(1–1) 1(1–5) 1(1–3)	3(3–10) 1(1–2) 1(1–5) 1(1–3)	0.542 0.774 0.321 0.416
**Treatment measures**				
Bystander CPR (n (%))	145(36.4)	71(35.7)	74(37.2)	0.835
CPR>15 min (n (%))	44(11.1)	22(11.1)	22(11.1)	1.000
CPR<15 min (n (%))	102(25.6)	50(25.1)	52(26.1)	0.909
Unwitnessed status (n (%))	14(3.5)	9(4.5)	5(2.5)	0.415
Enteral nutrition (n (%))	0(0)	0(0)	0(0)	/
Amiodarone (n (%))	9(2.3)	6(3.0)	3(1.5)	0.503
Dobutamine (n (%))	7(1.8)	3(1.5)	4(2.0)	1.000
Dopamine (n (%))	51(12.8)	28(14.1)	23(11.6)	0.549
Epinephrine (n (%))	31(7.8)	16(8.0)	15(7.5)	1.000
Statin (n (%))	6(1.5)	4(2.0)	2(1.0)	0.685

PSM, propensity score matching; CA, cardiac arrest; BMI, body mass index; HR, heart rate; SBP, systolic blood pressure; DBP, diastolic blood pressure; SpO2, saturation of peripheral oxygen; MI, myocardial infarction; AF, atrial fibrillation; CHF, chronic heart failure; CKD, chronic kidney disease; ARF, acute renal failure; RBC, red blood cell; WBC, white blood cell; HB, hemoglobin; BUN, blood urea nitrogen ; FBG, fast blood glucose; TyG-index, triglyceride-glucose index; TC, total cholesterol; HDL, high density lipoprotein; LDL, low density lipoprotein; APS, Acute Physiology Score; APACHE IV, Acute Physiology Age Chronic Health Evaluation IV; GCS, Glasgow Coma Scale; CPR, cardiopulmonary resuscitation

Lower group, TyG index<9.2; Higher group, TyG index ≥ 9.2

**Fig. 5:**
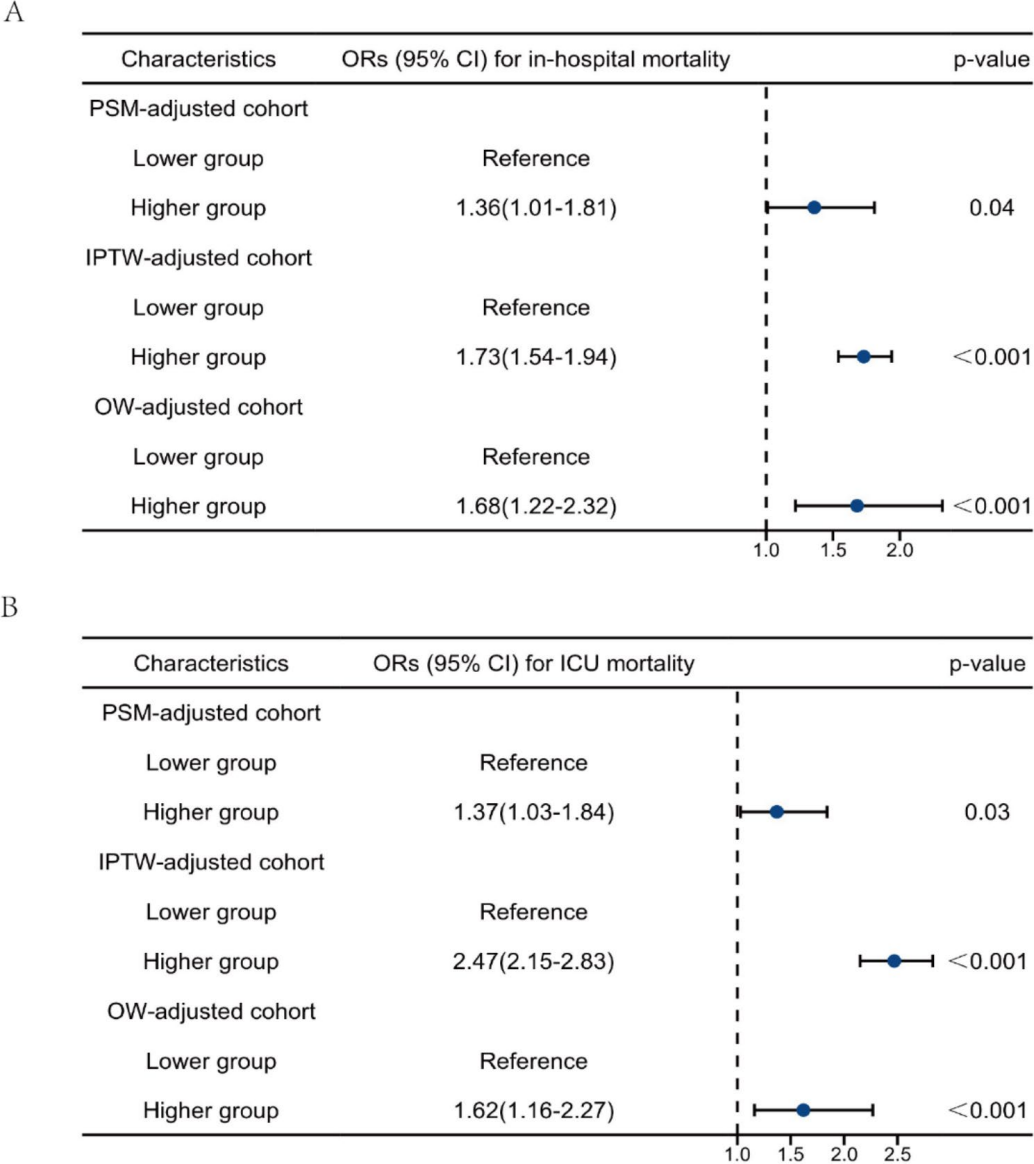
Logistic regression analysis to explore odds ratios of higher TyG index for in-hospital mortality and ICU mortality in PSM-, IPTW-, and OW-adjusted cohort. (A) Odds ratios for in-hospital mortality. (B) Odds ratios for ICU mortality. PSM, propensity score matching; IPTW, inverse probability of treatment weighting; OW, overlap weighting; TyG, triglyceride glucose index. Lower group, TyG index<9.2; Higher group, TyG index ≥ 9.2

The clinical outcomes in the original cohort, comprising the higher and lower TyG index groups, are presented in Table S7. Patients who were in post-CA and had a higher TyG index demonstrated a greater in-hospital mortality risk (42.0% vs. 32.3%) and ICU mortality risk (34.7% vs. 26.8%) as well as longer ICU LOS (3.50 (1.74–6.80) vs. 2.87 (1.48–6.33)) in the unadjusted cohort. The mortality rates were consistent in the PSM-adjusted cohort, but no significant differences were observed regarding LOS. (Table 7). Patients in the higher TyG group had significantly higher in-hospital mortality (41.7% vs. 31.9%) and ICU mortality (34.3% vs. 24.8%) rates post-PSM. A shorter LOS in the hospital and a longer LOS in the ICU were observed in the higher TyG index group, but no significant differences were found. Moreover, logistic regression for in-hospital mortality and ICU mortality was performed in the PSM-, IPTW-, and OW-adjusted cohorts (Fig. 5). Taken together, these results indicated that elevated TyG index is positively related to higher mortality rates in patients post-CA, even after adjusting by multiple statistical methods.

**Table 7:** Clinical outcomes between lower and higher TyG-index groups post PSM analysis

Clinical outcomes	Overall PSM-matched cohort(n = 398)	Lower TyG-index group (n = 199)	Higher TyG-index group (n = 199)	P-value
**Primary outcomes**				
In-hospital mortality (n (%))	182(45.7)	81(40.7)	101(50.8)	0.044
ICU mortality (n (%))	154(38.7)	67(33.7)	87(43.7)	0.040
**Secondary outcomes**				
Hospital LOS (days)	6.7(3.0-13.1)	8.0(3.3–14.1)	5.3(2.8–11.5)	0.139
ICU LOS (days)	3.6(1.7–6.9)	3.9(1.7–7.5)	3.3(1.8-6.0)	0.340

CA, cardiac arrest; ICU, intensive care unit; LOS, length of stay; TyG-index, triglyceride-glucose index; PSM, propensity score matching

Lower group, TyG index<9.2; Higher group, TyG index ≥ 9.2

### TyG index is negatively correlated with the neurological status of patients post-CA

Neurological outcomes are essential for patients post-CA. Hence, we further investigated the association between TyG index and GCS in these patients. As displayed in Table S8, significant differences of GCS, including motor, verbal and eyes scores were found between three tertiles of patients (P <0.001). Patients in the highest tertile of TyG index had a significant lower GCS than those in tertile 1 (4 [3–11] vs. 7 [3–15]). Similar results were observed in motor score (2 [1–6] vs. 5 [1–6]) and eyes score (1 [1–3] vs. 2 [1–4]).

Furthermore, we conducted correlation analyses to further explore the relationship between the TyG index and GCS using Pearson’s and Spearman’s methods. Interestingly, we found that TyG index was negatively correlated with GCS, including verbal, motor, and eyes scores (Table 8). Moreover, multiple linear regression confirmed that there was a significant linear correlation between TyG index and GCS (Table S4). Overall, our results suggest that there is a potential correlation between elevated TyG index and deteriorated neurological status in patients after CA.

**Table 8:** Relationship between TyG index and GCS in patients post-CA

Variables	Coef(a)	P-value	Coef(b)	P-value
GCS Verbal score Motor score Eyes score	-0.155 -0.162 -0.142 -0.135	<0.001 <0.001 <0.001 <0.001	-0.147 -0.152 -0.141 -0.128	<0.001 <0.001 <0.001 <0.001

TyG, triglyceride-glucose index; GCS, Glasgow Coma Scale; CA, cardiac arrest. a represents Pearson analysis, b represents spearman analysis

### TyG index is a risk factor for mortality in patients post-CA, independent of different modifiers

Subgroup analysis was performed based on age, BMI, sex, CHF, cardiogenic shock, MI, AF, and diabetes to evaluate the stability of the TyG index as a risk factor (Table S9). We found that the TyG index remained a risk factor for in-hospital mortality and ICU mortality independent of age, BMI, sex, CHF, cardiogenic shock, MI, and AF. Interestingly, in patients without diabetes, the TyG index seemed to be more closely associated with mortality risk than that in patients with diabetes. Moreover, no interactions between TyG and these modifiers were found, with all P for interactions being >0.05.

## Discussion

In this study, a significantly higher TyG index was observed in critically ill patients post-CA than in those who did not have CA. TyG index has a moderate discrimination ability to identify patients diagnosed with CA from the overall critically ill population. More importantly, the TyG index is highly related to in-hospital and ICU mortality risks in patients post-CA. Even after adjusting for many confounders using multiple statistical methods, the associations between TyG index and all-cause in-hospital or ICU mortality remained stable among these patients. Our study revealed the previously unrecognized role of the TyG index in CA, which extended the landscape of TyG index in cardiovascular disorders.

The importance of the TyG index in cardiovascular disorders has recently drawn extensive interest. The TyG index is a reliable indicator of the development of coronary artery calcification [6, 31] as well as the severity of coronary artery disease [32]. Moreover, the TyG index was positively associated with the risk of HF, hypertension, AF, and MI [2, 3, 33, 34]. The TyG index is also an independent risk factor for adverse prognosis in ICU, which is closely related to the increase in all-cause mortality in individuals who are critically ill [35]. The role of the TyG index as an indicator in cardiovascular disease could have the following possible explanation. The TyG index can reflect IR condition in a patient, while IR can promote the occurrence of cardiovascular diseases by increasing vascular stiffness and reducing NO bioavailability [36, 37].

Out-of-hospital SCD accounts for approximately 50% of all cardiovascular deaths [38, 39]. Although post-CA mortality declines over time, the in-hospital mortality rate remained as high as 57.8% in 2009 in the US and 66.4% in 2014 in the UK [40, 41]. In this study, a significantly higher in-hospital mortality (38.0% vs. 8.1%) and ICU mortality (30.8% vs. 3.9%) were observed in patients post-CA (Table S10). In approximately 50% of cases, SCA was most likely the first manifestation of cardiovascular diseases, with a survival rate of less than 20% [42]. That is, SCA is a life-threatening yet anonymous health problem. Although many effective predictors, including male sex, older age, diabetes mellitus, family history of CAD, are positively associated with the risk of CA occurrence [43], they are not effective enough to assess risks in a given patient owing to the relatively low occurrence rates [21]. In this study, we observed that the TyG index was greatly higher in individuals post-CA than in those who did not have CA in the critically ill population. More importantly, the TyG index had a greater discrimination ability to identify patients diagnosed with CA from the overall population than BMI, age, and sex. QT interval prolongation is a risk factor for sudden death in patients post-MI, and the risk of a malignant dysrhythmic event increased with a progressively longer QTc interval [44, 45]. Notably, an increased TyG index is related to QTc interval prolongation [46], which could partly explain why TyG is associated with the risk of CA occurrence. These findings indicate that the TyG index is expected to be a novel risk factor which is related to CA occurrence and still needs to be prospectively verified in the general population.

Although patients post-CA who were discharged from the hospital alive had greater 1-year survival rates than those hospitalized in the ICU for other causes [47], brain injury is emerging as an important health issue for the patients survived from CA [48] and irreversible brain injury further impaired the prognosis in these patients [49]. For those who are comatose post-CA, the Full Outline of Unresponsiveness and GCS are the most commonly used clinical scores [48]. Previous research revealed that GCS motor score 1 was an independent risk indicator for patients post-CA with OR = 2.00 (1.44–2.77) [28]. Hence, in this study, we included the GCS scores of patients on the first day of ICU admission and explored its correlation with the TyG index. Interestingly, we found that there was a significant negative correlation between the TyG index and GCS, including motor, eyes, and verbal scores, and this correlation might be linear. These findings indicated that the TyG index might have a potential association with the patient’s neurological outcomes, which requires further research in the future. In order to reduce the potential impacts of GCS on the prognosis, we balanced the GCS in the PSM cohort, and data showed that the higher TyG group still had significantly higher mortality rates. Additionally, the predictive ability of the FBG with the TyG index for mortality risk has improved in comparison to FBG alone (Table S11). Taken together, our results confirmed that TyG index might be a risk factor for higher mortality rates in patients post-CA, independent of the neurological status at ICU admission.

Many scores for the severity of illness in the ICU, including APACHE IV and APS scores, were proved to be a powerful risk stratification tool for the critically ill population [50]. However, the clinical application of most scoring systems is challenging in real-world scenarios because they necessitate the gathering of various physiological measures [51]. Interestingly, we found that the TyG index was positively associated with APACHE IV and APS scores in patients post-CA. These findings suggested that TyG index might be associated with the severity of illness in patients post-CA. Even after balancing APACHE IV and APS scores in the PSM cohort, patients post-CA with elevated TyG index still faced higher mortality risk. Regarding LOS in hospital and ICU, although a significantly increasing trend of LOS in hospital was observed with an increased tertile of the TyG index in this study, further correlation analysis showed no associations between the TyG index and LOS in hospital or ICU. Moreover, there was no significant difference in LOS between the high and low TyG populations, even after PSM. Therefore, no conclusive evidence was found in this study regarding the association between TyG and LOS in patients post-CA.

Researchers have developed several scoring systems to predict the prognosis of patients post-CA such as CRASS and CAHP score [19, 20]. These scoring systems demonstrated powerful predictive ability based on existing risk factors, including age, gender, witnessed status, bystander CPR, initial rhythm, the creatinine level, arterial pH, epinephrine use, delays from collapse to basic life support (BLS) and time from BLS to restoration of spontaneous circulation (ROSC) etc. No-flow time, low-flow time, and PaCO2 were also demonstrated to be early predictors of poor outcomes after OHCA [28]. In this study, we have included as many potential influencing factors as possible, which further increased the credibility of our results.

To our knowledge, this is the first research to explore the association between the TyG index and CA occurrence and prognosis based on a large-scale and multicenter database, which provides a new perspective for the role of the TyG index in cardiovascular disorders. The clinical significance of this study is to confirm a robust correlation between elevated TyG index and the occurrence and prognosis of CA. This means that patients with high TyG index can try to reduce it by adjusting lifestyle or treatment strategies, which may help reduce future incidence of CA and improve survival rates post- CA, at least at the population level. However, it should be noted that our findings did not confirm the causal relationship between the two, which must be verified further.

This study has the following limitations, which should be considered. First, this was a retrospective observational study; therefore, whether the elevation in the TyG index in this population is associated with an increased risk of CA requires validation through prospective trials.

It should also be noted that although we have adjusted as many potential confounders as possible, there are still some confounding factors that we have not considered in this study, such as no-flow time, low-flow time and time after the ROSC of patients post-CA, which have been shown to be risk factors for the prognosis of patients with CA [19, 52]. Moreover, the relationship between the TyG index and long-term events in individuals post-CA was not investigated in our study, which needs further exploration. Although this study provided evidence of associations between TyG index and CA, whether there is a causal relationship between them needs further research.

## Conclusion

TyG index is strongly associated with the occurrence and adverse clinical outcomes of CA in patients post-CA. Patients post-CA with elevated TyG index had higher risk of mortality. This study reported the unrecognized role of TyG index in the context of CA as an independent risk factor. Further prospective research is needed to verify our results.

## Electronic supplementary material

Below is the link to the electronic supplementary material.

Supplementary Material 1

## Data Availability

All the data are contained within the article.
