# Supplementary material for: Triglyceride-glucose index is associated with the occurrence and prognosis of cardiac arrest: a multicenter retrospective observational study
Source: Cardiovasc Diabetol. 2023 Jul 27;22:190. doi: 10.1186/s12933-023-01918-0 (PMC10375765; doi:10.1186/s12933-023-01918-0)
Supplement: Supplementary file 1 — Supplementary Material 1 [file 12933_2023_1918_MOESM1_ESM.docx]

**Table S1** AUCs of characteristics for identifying patients diagnosed with CA from the overall critically ill patients

| Characteristics | AUC | P-value |
| --- | --- | --- |
| Age | 0.482 | ＜0.001 |
| Sex | 0.528 | ＜0.001 |
| BMI | 0.529 | ＜0.001 |
| LDL | 0.417 | ＜0.001 |
| HDL | 0.418 | ＜0.001 |
| TC | 0.462 | ＜0.001 |

AUC, area under the curve; CA, cardiac arrest; BMI, body mass index; LDL, low density lipoprotein; HDL, high density lipoprotein; TC, triglyceride.

The P value represents the statistical difference compared to TyG index.

**Table S2** Baseline characteristics between survivors and non-survivors in patients post CA during hospital stay

|  | All patients with CA  (n=1021) | Survivors(n=642) | Non-survivors(n=379) | P-value |
| --- | --- | --- | --- | --- |
| **Demographic data** |  |  |  |  |
| Age (years） | 64(55-73) | 63(54-72) | 66(56-75) | 0.003 |
| Male (n (%)) | 648(63.5) | 429(66.8) | 219(57.8) | 0.005 |
| BMI | 29.0(25.0-34.0) | 29.3(25.3-33.9) | 28.4(24.2-34.4) | 0.672 |
| **Vital signs** |  |  |  |  |
| HR (/min) | 79(65-93) | 78(64-89) | 82(67-100) | ＜0.001 |
| SBP (mmHg) | 115(100-135) | 115(102-135) | 115(96-136) | 0.255 |
| DBP (mmHg) | 64(55-75) | 65(56-75) | 64(54-75) | 0.226 |
| Temperature (℃) | 36.6(34.6-37.1) | 36.7(36.0-37.1) | 36.0(33.3-37.0) | 0.013 |
| SpO2 | 99(95-100) | 99(96-100) | 99(95-100) | 0.001 |
| **Comorbidities** |  |  |  |  |
| MI (n (%)) | 249(24.4) | 181(28.2) | 68(17.9) | ＜0.001 |
| AF (n (%)) | 102(10.0) | 63(9.8) | 39(10.3) | 0.829 |
| CHF (n (%)) | 108(10.6) | 66(10.3) | 42(11.1) | 0.675 |
| CKD (n (%)) | 58(5.7) | 22(3.4) | 36(9.5) | ＜0.001 |
| ARF (n (%)) | 233(22.8) | 106(16.5) | 127(33.5) | ＜0.001 |
| Diabetes (n (%)) | 126(12.3) | 76(11.8) | 50(13.2) | 0.555 |
| Hypertension (n (%)) | 133(13.0) | 92(14.3) | 41(10.8) | 0.123 |
| cardiogenic shock (n (%)) | 122(11.9) | 71(11.1) | 51(13.5) | 0.272 |
| Non-shockable rhythm  (n (%)) | 255(25.0) | 203(31.6) | 52(13.7) | ＜0.001 |
| **Clinical indices** |  |  |  |  |
| RBC (M/mcl) | 4.1(3.5-4.6) | 4.1(3.6-4.6) | 3.9(3.3-4.6) | 0.016 |
| WBC (K/mcl) | 13.2(9.9-19.0) | 12.6(9.9-17.8) | 15.0(9.8-21.5) | 0.001 |
| HB(g/dL) | 12.1(10.2-13.8) | 12.2(10.4-13.8) | 11.7(9.7-13.5) | 0.004 |
| Platelet(K/mcl) | 197.0(148.0-248.5) | 202.5(157.0-250.3) | 188.0(137.0-243.0) | 0.263 |
| Creatinine (mg/dL) | 1.26(0.88-2.10) | 1.06(0.81-1.72) | 1.64(1.10-2.47) | ＜0.001 |
| BUN (mg/dL) | 22.0(15.0-34.0) | 19.0(14.0-29.0) | 28.0(20.0-40.0) | ＜0.001 |
| FBG (mg/dL) | 165.3(133.5-215.0) | 156.0(130.2-196.0) | 190.7(147.3-251.5) | ＜0.001 |
| Triglyceride (mg/dL) | 114.0(79.2-171.0) | 114.5(80.8-169.0) | 113.0(79.0-175.0) | 0.987 |
| Tyg-index | 9.20(8.72-9.69) | 9.13(8.68-9.57) | 9.28(8.81-9.86) | 0.001 |
| TC (mg/dL) | 136.0(107.4-166.3) | 143.0(115.4-174.0) | 123.0(93.0-152.0) | ＜0.001 |
| HDL (mg/dL) | 37.0(28.8-47.0) | 39.0(30.0-47.0) | 35.0(27.0-47.0) | 0.024 |
| LDL (mg/dL) | 71.0(47.6-96.9) | 78.0(55.8-101.7) | 60.5(39.1-83.4) | ＜0.001 |
| APS | 79(47-108) | 65(37-94) | 103(77-123) | ＜0.001 |
| APACHE IV | 91(60-122) | 76(47-104) | 116(89-137) | ＜0.001 |
| Arterial pH (mmHg) | 7.35(7.26-7.41) | 7.37(7.30-7.43) | 7.32(7.21-7.39) | ＜0.001 |
| PaCO2 (mmHg) | 39(33-46) | 39(34-45) | 40(33-47) | 0.107 |
| **Treatment measures** |  |  |  |  |
| Dobutamine (n (%)) | 19(1.9) | 11(1.7) | 8(2.1) | 0.640 |
| Dopamine (n (%)) | 110(10.8) | 59(9.2) | 51(13.5) | 0.037 |
| Epinephrine (n (%)) | 56(5.5) | 14(2.2) | 42(11.1) | ＜0.001 |
| Bystander CPR (n (%)) | 364(35.7) | 246(38.3) | 118(31.1) | 0.022 |
| CPR＞15 min (n (%)) | 89(8.7) | 42(6.5) | 47(12.4) | 0.002 |
| CPR＜15min (n (%)) | 278(27.2) | 205(31.9) | 73(19.3) | ＜0.001 |
| Unwitnessed status (n (%)) | 34(3.3) | 19(3.0) | 15(4.0) | 0.470 |
| Enteral nutrition (n (%)) | 5(0.5) | 2(0.3) | 3(0.8) | 0.366 |
| Amiodarone (n (%)) | 27(2.6) | 15(2.3) | 12(3.2) | 0.426 |
| Statin (n (%)) | 15(1.5) | 13(2.0) | 2(0.5) | 0.062 |

CA, cardiac arrest; BMI, body mass index; HR, heart rate; SBP, systolic blood pressure; DBP, diastolic blood pressure; SpO2, saturation of peripheral oxygen; MI, myocardial infarction; AF, atrial fibrillation; CHF, chronic heart failure; CKD, chronic kidney disease; ARF, acute renal failure; RBC, red blood cell; WBC, white blood cell; HB, hemoglobin; BUN, blood urea nitrogen ; FBG, fasting blood glucose; TyG-index, triglyceride-glucose index; TC, total cholesterol; HDL, high density lipoprotein; LDL, low density lipoprotein; APS, Acute Physiology Score; APACHE IV, Acute Physiology Age Chronic Health Evaluation IV.

| Variables | Coef(a) | P-value | Coef(b) | P-value |
| --- | --- | --- | --- | --- |
| Hospital LOS | -0.040 | 0.374 | -0.029 | 0.510 |
| ICU LOS | 0.028 | 0.536 | 0.057 | 0.198 |
| HDL | 0.006 | 0.884 | 0.015 | 0.734 |
| LDL | 0.079 | 0.074 | 0.082 | 0.064 |
| TC | 0.092 | 0.038 | 0.092 | 0.039 |
| APS | 0.091 | 0.040 | 0.105 | 0.018 |
| APACHE IV | 0.072 | 0.104 | 0.085 | 0.056 |
|  |  |  |  |  |

**Table S3** Relationship between TyG index and multiple continuous variables

LOS, length of stay; ICU, intensive care unit; TC, total cholesterol; HDL, high density lipoprotein; LDL, low density lipoprotein; APS, Acute Physiology Score; APACHE IV, Acute Physiology Age Chronic Health Evaluation IV.

a represents Pearson analysis, b represents spearman analysis.

**Table S4** Multiple linear regression analysis to explore relationship between TyG index and multiple continuous variables

| **Variables** | **β** | **t-value** | **P-value** |
| --- | --- | --- | --- |
| Hospital LOS | -0.072 | -1.647 | ＞0.9 |
| ICU LOS | -0.012 | -0.266 | 0.790 |
| APS | 0.102 | 2.700 | 0.007 |
| APACHE IV | 0.102 | 2.764 | 0.006 |
| GCS  Verbal score  Motor score  Eyes score | -0.155  -0.162  -0.142  -0.135 | -4.949  -5.157  -4.525  -4.277 | ＜0.001  ＜0.001  ＜0.001  ＜0.001 |

ICU, intensive care unit; TyG-index, triglyceride-glucose index; LOS, length of stay; APS, Acute Physiology Score; APACHE IV, Acute Physiology Age Chronic Health Evaluation IV; GCS, Glasgow Coma Scale.

**Table S5** Baseline characteristics between lower and higher TyG group in the original cohort

|  | Lower TyG group  (n=514) | Higher TyG group (n=507) | P-value |
| --- | --- | --- | --- |
| **Demographic data** |  |  |  |
| Age (years） | 65.0(56.0-75.0) | 63.0(54.0-71.0) | 0.002 |
| Male (n (%)) | 338(65.8) | 310(61.1) | 0.135 |
| BMI | 27.8(24.1-32.5) | 30.0(25.7-35.6) | 0.010 |
| **Vital signs** |  |  |  |
| HR (/min) | 78.0(64.0-91.0) | 80.0(67.0-95.0) | 0.012 |
| SBP (mmHg) | 116.0(100.0-135.0) | 114.0(100.0-134.0) | 0.776 |
| DBP (mmHg) | 65.0(56.0-75.0) | 64.0(55.0-75.0) | 0.188 |
| Temperature (℃) | 36.6(35.1-37.1) | 36.6(34.4-37.2) | 0.315 |
| SpO2 | 98.9(95.9-100.0) | 99.0(95.0-100.0) | 0.142 |
| **Comorbidities** |  |  |  |
| MI (n (%)) | 138(26.8) | 111(21.9) | 0.069 |
| AF (n (%)) | 48(9.3) | 54(10.7) | 0.532 |
| CHF (n (%)) | 60(11.7) | 48(9.5) | 0.264 |
| CKD (n (%)) | 29(5.6) | 29(5.7) | 1.00 |
| ARF (n (%)) | 96(18.7) | 137(27.0) | 0.002 |
| Diabetes (n (%)) | 57(11.1) | 69(13.6) | 0.253 |
| Hypertension (n (%)) | 70(13.6) | 63(12.4) | 0.578 |
| Cardiogenic shock (n (%)) | 52(10.1) | 70(13.8) | 0.082 |
| Non-shockable rhythm  (n (%)) | 144(28.2) | 111(21.8) | 0.021 |
| **Clinical indices** |  |  |  |
| RBC (M/mcl) | 3.99(3.44-4.55) | 4.12(4.63-3.49) | 0.061 |
| WBC (K/mcl) | 11.8(9.19-16.9) | 14.8(10.6-20.6) | 0.001 |
| Platelet (K/mcl) | 190.0(143.2-243.0) | 205.0(155.2-253.0) | 0.032 |
| HB (g/dL) | 11.9(10.1-13.5) | 12.2(10.2-13.9) | 0.024 |
| Creatinine (mg/dL) | 1.11(0.84-1.86) | 1.40(0.90-2.20) | 0.073 |
| BUN (mg/dL) | 20.0(14.0-32.0) | 23.0(16.0-36.0) | 0.184 |
| FBG (mg/dL) | 142.4(121.0-170.6) | 201.8(159.7-257.5) | <0.001 |
| Triglyceride (mg/dL) | 82.5(62.0-102.0) | 169.0(128.0-229.0) | <0.001 |
| Tyg-index | 8.72(8.39-8.97) | 9.70(9.40-10.1) | <0.001 |
| TC (mg/dL) | 129.0(102.0-158.6) | 141.0(113.0-175.0) | <0.001 |
| HDL (mg/dL) | 41.0(31.2-50.0) | 35.0(26.8-44.0) | <0.001 |
| LDL (mg/dL) | 67.7(45.0-93.1) | 76.4(52.0-99.4) | 0.003 |
| APS | 73.0(39.0-101.0) | 87.0(57.0-114.0) | <0.001 |
| APACHE IV | 86.0(52.0-114.0) | 100.0(68.0-124.7) | <0.001 |
| Arterial pH | 7.35(7.27-7.41) | 7.34(7.24-7.41) | 0.579 |
| PaCO2 (mmHg) | 40(33-48) | 39(33-45) | 0.014 |
| **Treatment measures** |  |  |  |
| Dobutamine (n (%)) | 11(2.1) | 8(1.6) | 0.645 |
| Dopamine (n (%)) | 57(11.1) | 53(10.5) | 0.763 |
| Epinephrine (n (%)) | 27(5.3) | 29(5.7) | 0.784 |
| Bystander CPR (n (%)) | 193(37.8) | 171(33.5) | 0.170 |
| CPR＞15 min (n (%)) | 41(8.0) | 48(9.4) | 0.440 |
| CPR＜15min (n (%)) | 153(29.9) | 125(24.5) | 0.058 |
| Unwitnessed status  (n (%)) | 19(3.7) | 15(2.9) | 0.601 |
| Enteral nutrition (n (%)) | 4(0.8) | 1(0.2) | 0.374 |
| Amiodarone (n (%)) | 12(2.3) | 15(2.9) | 0.566 |
| Statin (n (%)) | 7(1.4) | 8(1.6) | 0.802 |

CA, cardiac arrest; BMI, body mass index; HR, heart rate; SBP, systolic blood pressure; DBP, diastolic blood pressure; SpO2, saturation of peripheral oxygen; MI, myocardial infarction; AF, atrial fibrillation; CHF, chronic heart failure; CKD, chronic kidney disease; ARF, acute renal failure; RBC, red blood cell; WBC, white blood cell; HB, hemoglobin; BUN, blood urea nitrogen ; FBG, fasting blood glucose; TyG-index, triglyceride-glucose index; TC, total cholesterol; HDL, high density lipoprotein; LDL, low density lipoprotein; APS, Acute Physiology Score; APACHE IV, Acute Physiology Age Chronic Health Evaluation IV.

Lower TyG group, TyG index ≤ 9.20; Higher TyG group, TyG index ＞ 9.20;

**Table S6** Baseline characteristics in the matched cohort post IPTW and OW analysis

| IPTW-adjusted cohort | | | | OW-adjusted cohort | | | |  |  |
| --- | --- | --- | --- | --- | --- | --- | --- | --- | --- |
|  | Lower TyG group  (n=2087.3) | Higher Tyg group (n=1023.9) | P-value | Lower TyG group  (n=323.5) | Higher Tyg group (n=322.5) | P-value |  |  |  |
| **Demographic data** |  |  |  |  |  |  |  |  |  |
| Age (years） | 63.96 (12.04) | 59.68 (12.46) | 0.015 | 65.36 (14.20) | 61.13 (13.87) | <0.001 |  |  |  |
| Male (n (%)) | 0.75 (0.43) | 0.69 (0.46) | 0.614 | 0.67 (0.47) | 0.61 (0.49) | 0.038 |  |  |  |
| BMI | 102.62 (93.94) | 31.84 (12.06) | 0.119 | 29.10 (9.02) | 32.05 (9.95) | <0.001 |  |  |  |
| **Vital signs** |  |  |  |  |  |  |  |  |  |
| HR (/min) | 86.39 (19.47) | 76.81 (22.67) | 0.040 | 77.16 (19.84) | 82.39 (23.00) | <0.001 |  |  |  |
| SBP (mmHg) | 122.44 (20.70) | 111.32 (23.51) | 0.012 | 118.22 (26.13) | 117.28 (26.56) | 0.586 |  |  |  |
| DBP (mmHg) | 65.55 (16.84) | 64.44 (17.32) | 0.734 | 67.91 (17.65) | 65.21 (17.67) | 0.021 |  |  |  |
| Temperature (℃) | 35.51 (1.93) | 49.22 (25.46) | 0.076 | 35.86 (2.61) | 36.20 (5.17) | 0.269 |  |  |  |
| SpO2 | 98.27 (4.03) | 95.27 (10.17) | 0.086 | 97.35 (4.51) | 96.50 (7.76) | 0.049 |  |  |  |
| **Comorbidities** |  |  |  |  |  |  |  |  |  |
| MI (n (%)) | 233.0 (13.8) | 512.0 (12.9) | 0.883 | 95.3 (29.3) | 68.3 (2.1) | 0.004 |  |  |  |
| AF (n (%)) | 110.5 (6.5) | 267.0 (6.7) | 0.953 | 28.5 (8.8) | 34.5 (10.6) | 0.334 |  |  |  |
| CHF (n (%)) | 94.6 (5.6) | 226.8 (5.7) | 0.965 | 42.0 (12.9) | 30.0 (9.3) | 0.081 |  |  |  |
| CKD (n (%)) | 50.1 (3.0) | 182.5 (4.6) | 0.416 | 19.4 (6.0) | 19.4 (6.0) | 0.991 |  |  |  |
| ARF (n (%)) | 267.6 (15.8) | 1314.6 (33.1) | 0.085 | 54.3 (16.7) | 95.3 (29.4) | <0.001 |  |  |  |
| Diabetes (n (%)) | 129.2 (7.6) | 440.0 (11.1) | 0.442 | 35.2 (10.8) | 47.2 (14.6) | 0.090 |  |  |  |
| Non-shockable rhythm (n (%)) | 225.1 (13.3) | 1038.7 (26.2) | 0.220 | 101.6 (31.2) | 68.6 (21.2) | 0.001 |  |  |  |
| Hypertension (n (%)) | 145.4 (8.6) | 310.8 (7.8) | 0.852 | 46.7 (14.3) | 39.7 (12.2) | 0.349 |  |  |  |
| Cardiogenic shock (n(%)) | 108.8 (6.4) | 524.5 (13.2) | 0.118 | 30.6 (9.4) | 50.6 (15.6) | 0.005 |  |  |  |
| Clinical indices |  |  |  |  |  |  |  |  |  |
| RBC (M/mcl) | 3.75 (0.71) | 4.24 (0.75) | 0.002 | 3.96 (0.78) | 4.11 (0.82) | 0.004 |  |  |  |
| WBC (K/mcl) | 15.47 (24.88) | 17.60 (10.45) | 0.602 | 13.80 (9.73) | 17.53 (10.28) | <0.001 |  |  |  |
| Platelet (K/mcl) | 203.09 (74.63) | 201.73 (81.49) | 0.910 | 196.26 (77.27) | 212.93 (84.96) | 0.002 |  |  |  |
| HB (g/dL) | 10.70 (2.47) | 13.27 (2.65) | 0.002 | 11.75 (2.42) | 12.33 (2.54) | <0.001 |  |  |  |
| Creatinine (mg/dL) | 2.80 (1.81) | 1.82 (1.45) | 0.147 | 1.60 (1.36) | 1.86 (1.49) | 0.005 |  |  |  |
| BUN (mg/dL) | 45.05 (27.45) | 27.55 (18.23) | 0.120 | 26.48 (19.32) | 29.10 (18.73) | 0.036 |  |  |  |
| TC (mg/dL) | 122.14 (38.04) | 174.52 (47.15) | <0.001 | 129.93 (40.98) | 150.48 (46.05) | <0.001 |  |  |  |
| Arterial pH | 7.36 (0.11) | 7.31 (0.12) | 0.126 | 7.34 (0.12) | 7.33 (0.12) | 0.158 |  |  |  |
| HDL (mg/dL) | 20.29 (26.43) | 38.98 (22.32) | 0.134 | 43.95 (15.58) | 34.55 (14.18) | <0.001 |  |  |  |
| LDL (mg/dL) | 51.07 (38.75) | 83.65 (31.80) | 0.016 | 69.80 (34.95) | 80.66 (37.63) | <0.001 |  |  |  |
| APS | 92.22 (34.74) | 95.31 (36.69) | 0.770 | 69.96 (37.50) | 88.98 (37.29) | <0.001 |  |  |  |
| APACHE IV | 100.96 (33.71) | 104.73 (38.16) | 0.697 | 82.45 (38.85) | 99.54 (38.36) | <0.001 |  |  |  |
| Treatment measures |  |  |  |  |  |  |  |  |  |
| Bystander CPR (n (%)) | 353.7 (20.9) | 1301.1 (32.8) | 0.350 | 127.1 (39.1) | 105.1 (32.4) | 0.035 |  |  |  |
| CPR＞15 min (n (%)) | 78.0 (4.6) | 746.9 (18.8) | 0.043 | 25.3 (7.8) | 32.3 (10.0) | 0.243 |  |  |  |
| CPR＜15min (n (%)) | 277.7 (16.4) | 563.3 (14.2) | 0.744 | 102.3 (31.5) | 74.3 (22.9) | 0.004 |  |  |  |
| Unwitnessed status (n (%)) | 32.5 (1.9) | 52.8 (1.3) | 0.531 | 12.3 (3.8) | 8.3 (2.6) | 0.279 |  |  |  |
| Enteral nutrition (n (%)) | 4.6 (0.3) | 1.9 (0.0) | 0.093 | 3.5 (1.1) | 0.5 (0.1) | 0.034 |  |  |  |
| Amiodarone (n (%)) | 20.9 (1.2) | 98.3 (2.5) | 0.246 | 7.7 (2.4) | 10.7 (3.3) | 0.403 |  |  |  |
| Dobutamine (n (%)) | 15.9 (0.9) | 33.3 (0.8) | 0.867 | 7.9 (2.4) | 4.9 (1.5) | 0.316 |  |  |  |
| Dopamine (n (%)) | 104.1 (6.2) | 515.2 (13.0) | 0.193 | 37.5 (11.5) | 33.5 (10.3) | 0.563 |  |  |  |
| Epinephrine (n (%)) | 85.9 (5.1) | 195.7 (4.9) | 0.960 | 16.0 (4.9) | 18.0 (5.6) | 0.666 |  |  |  |
| Statin (n (%)) | 13.7 (0.8) | 61.2 (1.5) | 0.430 | 4.1 (1.3) | 5.1 (1.6) | 0.682 |  |  |  |

TyG, triglyceride-glucose index; CA, cardiac arrest; BMI, body mass index; HR, heart rate; SBP, systolic blood pressure; DBP, diastolic blood pressure; SpO2, saturation of peripheral oxygen; MI, myocardial infarction; AF, atrial fibrillation; CHF, chronic heart failure; CKD, chronic kidney disease; ARF, acute renal failure; RBC, red blood cell; WBC, white blood cell; HB, hemoglobin; BUN, blood urea nitrogen; TC, total cholesterol; HDL, high density lipoprotein; LDL, low density lipoprotein; APS, Acute Physiology Score; APACHE IV, Acute Physiology Age Chronic Health Evaluation IV; CPR, cardiopulmonary resuscitation.

Lower TyG group, TyG index ≤ 9.20; Higher TyG group, TyG index＞9.20.

**Table S7** Clinical outcomes between lower and higher TyG groups in the original cohort

| **Clinical outcomes** | Lower TyG group  (n=514) | Higher Tyg group (n=507) | P-value |
| --- | --- | --- | --- |
| **Primary** **outcomes** |  |  |  |
| In-hospital mortality (n (%)) | 166(32.3) | 213(42.0) | 0.001 |
| ICU mortality (n (%)) | 138(26.8) | 176(34.7) | 0.007 |
| **Secondary outcomes** |  |  |  |
| Hospital LOS (days) | 5.90(2.96-11.9) | 5.93(2.97-12.4) | 0.35 |
| ICU LOS (days) | 2.87(1.48-6.33) | 3.50(1.74-6.80) | 0.015 |

TyG, triglyceride-glucose index; ICU, intensive care unit; LOS, length of stay.

**Table S8** GCS of patients post-CA grouped according to TyG index tertile

| **Clinical outcomes** | Tertile 1  (n=340) | Tertile 2  (n=341) | Tertile 3  (n=340) | P-value |
| --- | --- | --- | --- | --- |
| **Primary** **outcomes** |  |  |  |  |
| GCS  Verbal score  Motor score  Eyes score | 7(3-15)  1(1-5)  5(1-6)  2(1-4) | 7(3-14)  1(1-5)  2(1-4)  4(1-6) | 4(3-11)  1(1-2)  2(1-6)  1(1-3) | ＜0.001  ＜0.001  ＜0.001  ＜0.001 |

GCS, Glasgow Coma Scale; CA, cardiac arrest; TyG, triglyceride-glucose index.

**Table S9** Association between TyG-index and clinical outcomes stratified by age, BMI, gender, CHF, cardiogenic shock, MI, AF, diabetes in patients post-CA.

|  | Number of patients | OR for  in-hospital mortality | OR for  ICU mortality | P-values for  interaction |
| --- | --- | --- | --- | --- |
| **Age** |  |  |  | 0.320 |
| ＞65 | 458 | 1.59(1.21-2.10) | 1.55(1.17-2.05) |  |
| ≤65 | 563 | 1.28(1.02-1.61) | 1.34(1.05-1.71) |  |
| **BMI** |  |  |  | 0.465 |
| ≥29 | 504 | 1.35(1.04-1.74) | 1.34(1.02-1.76) |  |
| ＜29 | 517 | 1.57(1.22-2.02) | 1.59(1.23-2.06) |  |
| **Gender** |  |  |  | 0.267 |
| Male | 648 | 1.31(1.04-1.64) | 1.30(1.03-1.66) |  |
| Female | 373 | 1.50(1.13-1.99) | 1.55(1.16-2.07) |  |
| **CHF** |  |  |  | 0.425 |
| Yes | 108 | 1.89(1.07-3.37) | 2.06(1.16-3.65) |  |
| No | 913 | 1.36(1.13-1.64) | 1.35(1.11-1.64) |  |
| **Cardiogenic shock** |  |  |  | 0.054 |
| Yes  No | 122  899 | 2.24(1.29-3.90)  1.31(1.09-1.58) | 1.90(1.09-3.32)  1.36(1.11-1.65) |  |
| **MI** |  |  |  | 0.133 |
| Yes | 249 | 2.08(1.33-3.25) | 2.11(1.34-3.33) |  |
| No | 772 | 1.30(1.07-1.58) | 1.31(1.07-1.60) |  |
| **AF** |  |  |  | 0.173 |
| Yes | 102 | 2.06(1.09-3.93) | 2.18(1.09-4.34) |  |
| No | 919 | 1.35(1.12-1.62) | 1.39(1.15-1.68) |  |
| **Diabetes** |  |  |  | 0.260 |
| Yes  No | 126  895 | 1.05(0.97-1.03)  1.46(1.21-1.78) | 1.05(0.97-1.03)  1.48(1.21-1.81) |  |

TyG-index, triglyceride-glucose index; ICU, intensive care unit; BMI, body mass index; CHF, chronic heart failure; MI, myocardial infraction; AF, atrial fibrillation; CA, cardiac arrest.

**Table S10** Clinical outcomes between critically ill patients without CA and with CA

| **Clinical outcomes** | All critically ill patients  (n=24689) | Patients without CA (n=23668) | Patients with CA (n=1021) | P-value |
| --- | --- | --- | --- | --- |
| **Primary** **outcomes** |  |  |  |  |
| In-hospital mortality (n (%)) | 2303(9.3) | 1915(8.1) | 388(38.0) | ＜0.001 |
| ICU mortality (n (%)) | 1234(5.0) | 920(3.9) | 314(30.8) | ＜0.001 |
| **Secondary outcomes** |  |  |  |  |
| Hospital LOS (days) | 4.6(2.5-8.8) | 4.6(2.5-8.7) | 5.9(3.0-12.1) | ＜0.001 |
| ICU LOS (days) | 1.7(1.0-3.2) | 1.7(1.0-3.1) | 3.2(1.6-6.6) | ＜0.001 |

ICU, intensive care unit; CA, cardiac arrest; LOS, length of stay.

**Table S11** The NRI and IDI for the incremental values of TyG index for mortality risk

|  | IDI | P-value | NRI | P-value |
| --- | --- | --- | --- | --- |
| FBG | Reference |  |  |  |
| FBG+TyG index for in-hospital mortality | 0.041(0.027-0.054) | ＜0.001 | 0.079(0.044-0.118) | 0.017 |
| FBG+TyG index for ICU mortality | 0.040(0.026-0.054) | ＜0.001 | 0.091(0.050-0.134) | 0.020 |

FBG, fasting blood glucose; TyG-index, triglyceride-glucose index; NRI, net reclassification index; IDI, integrated discrimination improvement.
